# A Sustainable Method for Removal of the Full Range of Liquid and Solid Hydrocarbons from Water Including Up‐ and Recycling

**DOI:** 10.1002/advs.202302495

**Published:** 2023-10-09

**Authors:** Henrik Gaß, Marco Sarcletti, Lukas Müller, Sabine Hübner, Tadahiro Yokosawa, Hyoungwon Park, Thomas Przybilla, Erdmann Spiecker, Marcus Halik

**Affiliations:** ^1^ Organic Materials & Devices Institute of Polymer Materials Friedrich‐Alexander‐University Erlangen Nürnberg 91058 Erlangen Germany; ^2^ Institute of Micro‐ and Nanostructure Research (IMN) & Center for Nanoanalysis and Electron Microscopy (CENEM) Friedrich‐Alexander‐University Erlangen‐Nürnberg 91058 Erlangen Germany

**Keywords:** environmental remediation, hydrocarbons, recycling, self‐assembled monolayers, superparamagnetic iron oxide nanoparticles, upcycling, water contamination

## Abstract

Beyond their CO_2_ emittance when burned as fuels, hydrocarbons (HCs) serve as omnipresent raw materials and commodities. No matter if as liquid oil spills or the endless amounts of plastic roaming the oceans, HCs behave as persistent pollutants with water as main carrier to distribute. Even if their general chemical structure [‐(CH_2_)*_n_*‐] is quite simple, the endless range of *n* leads to contaminations of different appearances and properties. A water remediation method based on superparamagnetic iron oxide nanoparticles (SPIONs) modified with self‐assembled monolayers of alkyl phosphonic acid derivatives is presented. These molecules enable the SPIONs to non‐covalently bind HCs, independently from the molecular weight, size and morphology. The attractive interaction is mainly based on hydrophobic and Coulomb interaction, which allows recycling of the SPIONs. The superparamagnetic core allows a simple magnetic collection and separation from the water phase which makes it a promising addition to wastewater treatment. Agglomerates of collected plastic “waste” even exhibit superior adsorption properties for crude oil, another hydrocarbon waste which gives these collected wastes a second life. This upcycling approach combined with presented recycling methods enables a complete recycling loop.

## Graphical abstract

A special surface modification can turn rust into smart rust. This enables agglomeration with basic hydrocarbons from liquid alkanes to polyethylene microplastics. These aggregates can be magnetically removed from the water and upcycled for oil adsorption. The nanoparticles can be separated from the organic contaminants for multiple reuse, enabling a complete recycling loop.

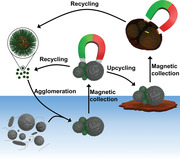

## 1 Introduction

Despite all attempts to attenuate the climate change that is driven by CO_2_‐emission from burning fossil fuels, hydrocarbons (HCs) still serve as propellent for global economy. The world production of crude oil as main source of liquid fuels still exceeds 4200 Mt (million tons) added by >4000 billion m^3^ of natural gas in 2021.^[^ ^1^ ^]^ Besides of direct use as fuels, the HCs also serve as major source for basic chemical compounds and common materials such as polymers – called plastics – with 391 Mt production in 2021.^[^ ^2^ ^]^ Excepting the gaseous HCs that emit to atmosphere, the production, the transportation and the use of such enormous amounts of liquid and solid HCs automatically lead to environmental contaminations of soil, air and in particular water.^[^ ^3^, ^4^, ^5^, ^6^, ^7^ ^]^

Thereby, the simple chemical structure of HCs that are composed only of hydrogen saturated sp^3^‐carbon atoms of various numbers, represent the majority of such pollutions. They range from liquid fuels (e.g., gasoline, diesel) over waxes or soft oligomer additives up to high molecular weight commodity polyethylene (PE): The PE‐family alone (e.g., low density‐PE, high density‐PE) represents 27% market share of all plastics in Europe.^[^ ^2^ ^]^ The HCs (with the general structure: *–(CH_2_)_n_–*) represent contaminations that bridge aggregate states (liquid, solid), that appear in different shapes (droplets, flakes, particles, foils) and that exist in different morphologies (amorphous, semi‐crystalline, cross‐linked). All these variations behave as a function of the number of repeating unit *n* or more simplified on the number of carbon atoms (C_5_ → C_∞_ for liquid to solid cross‐linked PE) and their chemical manufacturing. However, all HCs share one common property. The simple composition of only C‐C or C‐H single bonds makes them chemically rather inert. That leads on one hand to favorable properties as long term storage stability of fuels or light‐weight nature and melt processability for plastics. On the other hand, as soon as they are released into the environment, the persistence prevents fast biological or oxidative degradation.^[^ ^8^ ^]^ As consequence, spilled HCs roam around for decades and tend to distribute.^[^ ^7^, ^9^, ^10^ ^]^ Crude oil and refined fuels serve as dramatic long term impacts in ecosystems when spilled accidentally in large quantities^[^ ^11^, ^12^ ^]^ or accumulate locally over time.^[^ ^13^ ^]^ The solid plastic waste is distributed around the globe and exposed to various chemical and mechanical degradation mechanisms. A combination of these leads to a gradual degradation from macro‐ and meso‐ to micro‐ and finally to nanoplastics.^[^ ^14^, ^15^ ^]^ The fragmentation leads to a dramatic increase in the number of debris particles. Therefore, one small plastic piece of PE with a size of a sugar cube could fragment over time into 5 trillion (5∙10^12^) nanoplastic particles of 100 nm diameter. Simultaneously, the surface area of the plastic waste increases. That makes nanoplastics not only a potential contaminant that triggers inflammation in organisms but also a prospective carrier for accumulated toxins.^[^ ^16^, ^17^, ^18^ ^]^

Eventually, these potentially hazardous HCs will end up in the aquatic environment, where they are primarily distributed.^[^ ^19^, ^20^ ^]^ The interruption of transport by rivers could lead to a realistic approach to tackle further drain of HC waste to the oceans because the remediation from oceans with app. 1.3 billion km^3^ water volume occurs much more challenging. This means that a simple, reliable method to remove all kind of HCs from water with which sewage plants around the world could be equipped is highly demanded. In order to achieve sustainable water cleaning, such a method has to meet additional requirements in terms of efficiency, scalability, low‐cost, nontoxicity and the capability of recycling of materials. Several approaches are used as state‐of‐the‐art to handle liquid HC contaminations, ranging from physical separation, mechanical collection or dispersion.^[^ ^21^ ^]^ The concepts for removing micro‐ and nanoplastics are limited so far. It is possible to remove portions of the microplastics by skimming and flocculation.^[^ ^22^ ^]^ However, the smaller pieces are still a huge challenge for today's wastewater treatment plants.^[^ ^23^, ^24^ ^]^ As emerging contaminants, the focus is still on the quantification and the qualification of the dimension of the waste problem and on the ecological impact.^[^ ^25^, ^26^, ^27^, ^28^, ^29^ ^]^ Thereby, polystyrene (PS) is investigated most intensively due to the accessibility of artificial model systems in various sizes. For persistent contaminations as HCs (e.g., PE), that could not degrade in the ecosystem directly, the adsorption on suitable surfaces with subsequent removal from the water phase followed by re‐ and/or upcycling provides a potential method in wastewater treatment.

Superparamagnetic iron oxide nanoparticles (SPIONs) are widely investigated, e. g. as magnetic carrier in medical applications.^[^ ^30^, ^31^, ^32^ ^]^ They not only benefit from their simple accessibility and flexibility to equip their surface with functional features but also on their non‐toxicity.^[^ ^33^, ^34^ ^]^ In wastewater treatment, SPIONs have shown the ability to adsorb various liquid organic pollutants as alkanes and aromatic compounds, glyphosate or polychlorinated biphenyls (PCBs) by applying differently functionalized SPION surfaces that range from bare OH‐termination to hydrophobic decoration with organic molecules up to more complex shell‐by‐shell double‐layer systems.^[^ ^35^, ^36^, ^37^, ^38^ ^]^ The successful water treatment was shown for artificial and real water systems.^[^ ^38^, ^39^ ^]^ Recently, SPIONs that are tuned toward charged surfaces have been shown to collect nanoplastics (below 1 µm) of polystyrene and melamine resin from artificial and real water sources.^[^ ^40^, ^41^ ^]^

**Figure 1:**
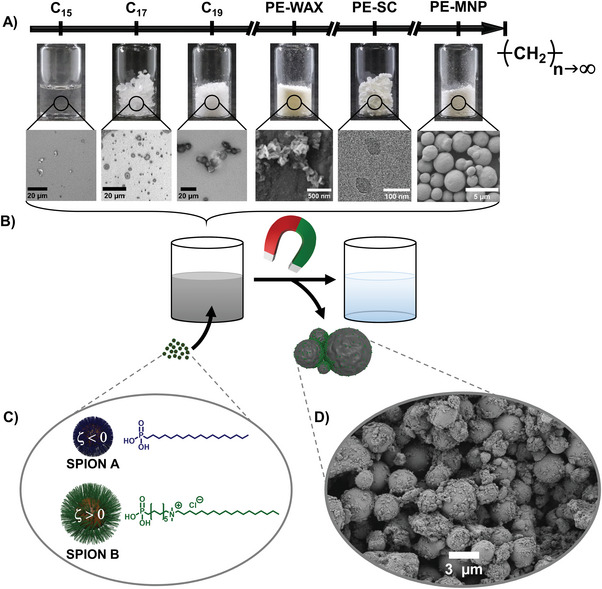
Schematic depiction of the range of used hydrocarbons, ranging from liquid C_15_ over polyethylene wax (PE‐WAX) and polyethylene single crystals (PE‐SC) to cross‐linked polyethylene micro‐ and nanoplastics (PE‐MNP) including microscopic images A), the removal procedure B), the used particle systems including the molecular structure of the surfactant C) and a SEM image of superparamagnetic iron oxide nanoparticle (SPION)‐polyethylene agglomerate D).

Here, we demonstrate a universal remediation concept to remove the full range of HCs from C_15_ to C_∞_ (from n‐pentadecane to crosslinked PE) magnetically from water (s. **Figure** **1**B). The collected HC pollutants comprise compounds from molecular scale up to microparticles (1.8 nm to 10 µm), from liquid to solid and from amorphous to single crystalline that can be collected with only one sorbent system (s. Figure 1A and **Table** **1**). The catching effect is based on non‐covalent interaction of HCs with functionalized SPIONs. The superparamagnetic nanoparticle core (maghemite – γ‐Fe_2_O_3_) of ≈10 nm diameter provides a large interaction surface and acts as a carrier for simple magnetic collection from water after a HC‐SPION hybrid is formed. Self‐assembled monolayers (SAMs) of organic molecules provide the functionality of SPION surface to introduce van‐der‐Waals and electrostatic interaction motifs as well as to provide hydrophobicity to attract the HCs compared to the “wastewater” phase. In case of small molecular liquid HCs, the HC‐SPION hybrids could be understood as corona‐like systems with solid SPIONs or small aggregates of those in the center of the liquid HC droplet.^[^ ^39^ ^]^ For solid HCs the collected hybrids occur as aggregates where the SPIONs act as glue between the plastics particles (s. Figure 1D).^[^ ^40^ ^]^ In any case, the content of superparamagnetic γ‐Fe_2_O_3_ enables the simple magnetic remediation with an external magnet. The non‐covalent character of HC‐SPION interaction ensures reversibility of HC‐binding to the SPION surface as key toward simple recycling concepts. Independent from the aggregate state of the HC, the SPIONs can be recycled efficiently and reused to collect liquid or solid HCs. A cascade upcycling approach, which applies already collected aggregates of SPIONs and PE micro‐/nanoplastics (PE‐MNP) to remove liquid HCs (e.g., crude oil) afterwards, leads to an increased remediation performance by more than 50% compared to oil collection with SPIONs only. The collected micro‐/nanoplastic‐SPION “waste” appears as valuable material in magnetic wastewater cleaning from liquid HCs (waste x waste = waste^2^).

**Table 1:** Overview of the used hydrocarbons.

	Name	# C atoms	Molecular weight [g mol^−1^]	Primary size [nm]	Aggregate phase
C15	n‐pentadecane	15	212.42	1.78	liquid
C17	n‐heptadecane	17	240.47	2.04	intermediate
C19	n‐nonadecane	19	268.53	2.29	solid
PE‐WAX	polyethylene wax	32‐43	450‐600	1219 ± 350.4	solid
PE‐SC	polyethylene single crystals	42 766	3 206 000	49.61 ± 5.25	solid
PE‐MNP	polyethylene micro‐ and nanoplastics	“∞”	“∞”	740 – 4990	solid

## 2 Results

### 2.1 Characterization of SPION Systems

In our study, we have used two core‐shell SPION sorbent systems (A and B) that are built on the same commercial SPION core but differ in the shell composition (s. Figure 1C). The shell molecules self‐assemble as a monolayer via phosphonic acid anchor groups and bind covalently toward very robust functional SPIONs.^[^ ^34^, ^42^ ^]^ This ensures no chemical degradation of the sorbent system as requirement for efficient reusability. Commercial SPIONs were functionalized with n‐octadecylphosphonic acid (PAC_18_) to SPION A (s. Figure 1). The long alkyl chain of PAC_18_ introduces hydrophobicity and van‐der Waals motifs as the main interaction features with non‐polar HCs.^[^ ^39^ ^]^ Additionally, the ζ‐potential of the particles is changed from a slightly positive for non‐functionalized SPIONs (ζ  =  +9.04 ± 2.26 mV) to more negative values (ζ = – 13.7 ± 6.9 mV, s. Figure S1C, Supporting Information). Second, the same core nanoparticles were functionalized with (12‐dodecylphosphonic acid)‐N,N‐dimethyl‐N‐octadecyl ammonium chloride (PAC_12_NC_18_) to yield SPION B (s. Figure S1A+B, Supporting Information). Here, the ζ‐potential was increased (ζ = + 23.0 ± 8.5 mV) during functionalization since PAC_12_NC_18_ carries a positive true charge that is buried in its backbone. This introduces electrostatic interaction as another strong attraction motif between SPION B and HCs since n‐alkane droplets as well as PE typically show a negative ζ‐potential in neutral water.^[^ ^43^, ^44^, ^45^ ^]^ The long upper tail of the SAM contributes to hydrophobicity of SPION B. The characterization of SPION A and B by Fourier‐transformed infrared spectroscopy, thermogravimetric analysis (TGA) and pH‐dependent ζ‐potential measurements is provided in Figure S1 (Supporting Information). Overall, the functionalization serves two different alkyl‐terminated hydrophobic SPION systems where SPION A exhibits a negative surface potential and SPION B a positive surface potential respectively.

### 2.2 Removal of Hydrocarbons

In order to quantify the remediation of solid and liquid HCs, SPIONs A and B were added independently to corresponding aqueous samples of 6 different HCs (s. Figure 1A). As small molecule HCs, we have chosen liquid n‐pentadecane (C_15_, M = 212.42 g mol^−1^), n‐heptadecane (C_17_, M = 240.47 g mol^−1^), with a melting temperature of 21.7 °C and solid n‐nonadecane (C_19_, M = 268.53 g mol^−1^).^[^ ^46^ ^]^ With these samples, we draw the attention to the transition in aggregate state of HCs that is concomitant with the morphological appearance from small droplets for liquid HCs to larger waxy flakes for solids. Smaller HCs (e.g., n‐heptane or iso‐octane) have been studied recently.^[^ ^39^ ^]^ As solid polymer samples PE variants with increasing molecular weight were investigated, namely a PE‐based wax (PE‐WAX, M  =  450‐600 g mol^−1^), tiny PE single crystals (PE‐SC, M  =  3 206 000 g mol^−1^) and cross‐linked PE micro‐/nanoplastics (PE‐MNP, M  =  “∞” g mol^−1^). This series represents samples of solid HCs with different morphologies (waxy, single crystalline and cross‐linked) and sizes ranging between 45 nm and 10 µm. Size, ζ‐potential and FTIR analysis are shown in Figure S2 (Supporting Information). The HCs were dispersed in water according to the method section. SPIONs were added and after magnetic collection of the HC‐SPION hybrids the hydrocarbon collection capability (HCC) was determined. HCC expresses either the collected amount of HCs related to weight (how much HC is collected per added SPION – [g/g]) or to number (how many HC precipitations (C_15_ – C_19_, PE‐WAX), crystals (PE‐SC) or spheres (PE‐MNP) per gram of SPION [#/g]). The latter is of particular interest due to the different size ranges of the HC species. The HCC values were determined either by gas chromatography‐mass spectrometry for small molecule HCs or by TGA for wax and plastic samples.

**Figure 2:**
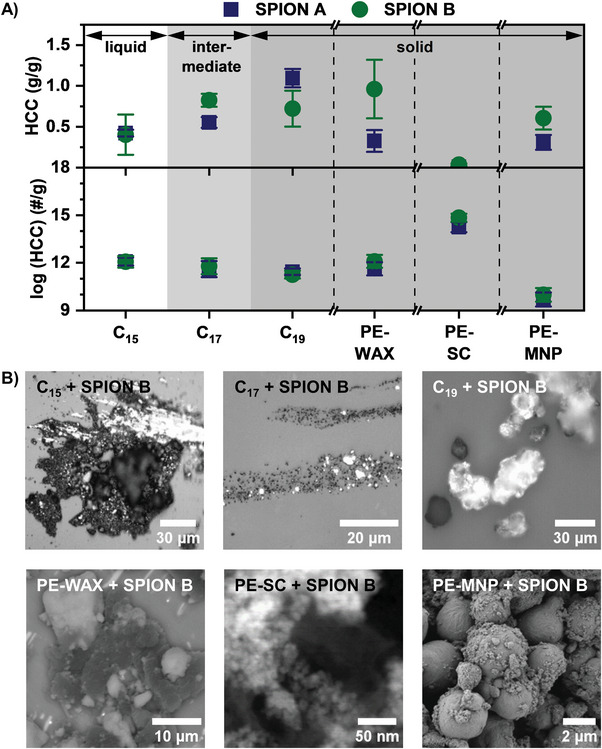
A) The HCC (g/g, top) for n‐alkanes show an increasing rate for higher molecular weight and therefore, a more solid state. The HCC of PE‐WAX, PE‐SC, and PE‐MNP is consistently higher for SPION B. The conversion to ER_mass_ (#/g, bottom) reveals the potential to adsorb up to one quadrillion PE‐SCs with only 1 g SPIONs. B) Reflected light microscopy images for C_15_, C_17_, and C_19_, SEM images for PE‐WAX and PE‐MNP as well as a transmission electron microscopy (TEM) images for PE‐SC of the formed agglomerates with SPION B are shown. A deeper SEM and TEM analysis is provided in the supplementary information.

The remediation results are collected in **Figure** **2**A. A successful magnetic remediation is obtained with both SPION systems and independent of aggregate state of the HCs. In average, SPIONs A and B are able to remove between 0.5 and 1 g HCs per gram of SPION with one collection cycle. Only the very small PE‐SC exhibits special results. Here, the collection performance seems to be virtually impaired when considering HCC [g/g] whereas HCC [#/g] indicates a markedly good performance. A deeper look in the experimental results of the remediation experiments indicates some general conclusions addressing size, morphology and surface composition of HCs related to the two different SPION systems.

The transition from liquid to solid HCs leads to slightly higher HCC values from 0.42 ± 0.04 g/g for C_15_ to 1.09 ± 0.11 g/g for C_19_ when using SPION A, exemplarily (s. Figure 2A). The number weighted HCC [#/g] decreases in parallel. We address this effect to the change in morphology from small liquid droplets to waxy flakes with larger size variation (s. Figure 2B). The change in state of aggregate causes a different interaction mechanism between SPION and the corresponding HCs. While liquid HCs tend to surround the solid SPION and their HCC occurs quite independent from the chemical structure and viscosity,^[^ ^39^ ^]^ solid HCs depend on particle‐particle interaction with the SPIONs via attractive surfaces.^[^ ^40^ ^]^

**Figure 3:**
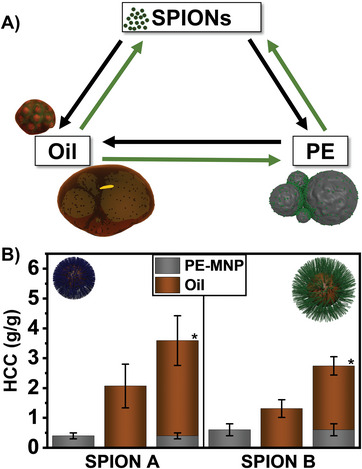
A schematic depiction A) shows a possible up‐ and recycling loop: The initial functionalized SPIONs can be reached again regardless of the type of amorphous hydrocarbon. In addition, the collected plastic can serve as a sorbent for liquid hydrocarbons. Successively collecting PE‐MNP and crude oil show superior adsorption capabilities compared to only using functionalized SPIONs due to the accessible porous structure of the PE‐SPION agglomerates B). Columns marked with a star (*) are composed of 2 differently determined extraction values.

A difference of SPION A and B in remediation performance toward small molecule HCs is not pronounced. For liquid C_15_, both exhibit similar results while for C_17_ SPION B and for C_19_ SPION A performs more efficiently. These results are surprising as further experiments have shown that typically SPION A outperforms SPION B in collection potential of liquid HCs from the water surface (s. **Figure** **3**B). In the water column the dispersibility of the SPIONs plays a critical role. The positive true charge and the resulting larger absolute value in ζ‐potential for SPION B in comparison to SPION A facilitates the dispersibility and therefore, provides a higher surface area for the liquid hydrocarbon to interact with. This may compensate the expected higher interaction potential of SPION A due to the long, non‐charged and non‐polar alkyl chain. Furthermore, the surface charge seems to play the critical role for C_17_: the electrostatic attraction between negatively charged alkane precipitations and positively charged SPION increases the HCC of SPION B compared to SPION A. This was proven before for nanoplastics.^[^ ^40^ ^]^ In contrast, SPION A outperforms SPION B in collecting C_19_, which contradicts an electrostatic interaction‐based explanation. Therefore, the difference in collection potential may relate to the different morphological appearance of C_17_ and C_19_. For the higher molecular weight PE variants, namely PE‐WAX, PE‐SC and PE‐MNP, the general feasibility as well as the superior extraction behavior of positively charged SPION B is shown in Figure 2A. While SPION B shows a removal rate of 0.60 ± 0.14 g/g PE‐MNP, SPION A only exhibits 0.31 ± 0.09 g/g. For PE‐WAX the difference is even more pronounced: SPION B (0.96 ± 0.36 g/g) shows three times the collection values as SPION A (0.33 ± 0.13 g/g). The collected SPION‐HC agglomerates of all variants are shown in form of microscopic images from scanning electron microscopy (SEM) and transmission electron microscopy (TEM) in Figure 2B exemplarily for SPION B. Additionally, secondary electron (SE) images and energy‐dispersive X‐ray spectroscopy (EDXS) maps acquired by SEM are shown in Figures S3 and S4 (Supporting Information), which allow to distinguish between Fe and C in the PE‐WAX and PE‐SC SPION hybrids.

However, the thermogravimetric quantification method is facing limitations for very small fragment species, as seen for PE‐SC. The tiny and light‐weighted single crystals saturate the SPION surface without contributing significantly to the mass of the PE‐SPION agglomerates. Nevertheless, the success of magnetic collection of SPION‐PE‐SC agglomerates is seen in an analysis by energy dispersive X‐ray spectroscopy with a scanning transmission electron microscope (STEM‐EDX) (s. Figure S4, Supporting Information). Here, the aforementioned conversion from HCC [g/g] to HCC [#/g] simplifies the comprehension of the removal efficiency. This transformation is mainly determined by the polymer size, beside the HCC: the smaller the PE droplets, precipitations or spheres, the higher the number extraction. Therefore, the measured value is comparably low regarding mass but high regarding numbers. This conversion clearly shows the potential of our modified SPIONs, which are capable of collecting up to a quadrillion (10^15^) PE micro/nano‐fragments using only 1 g of SPION.

### 2.3 Re‐ & Upcycling

As resources are limited and sustainability has become of highest importance, recycling is a crucial factor in future waste management. Therefore, our SPION system is designed to be recyclable for multiple use. To prove this, the collected HCs have to be separated from the SPIONs. The liquid HCs can be easily washed off the particles with an according solvent, such as hexane, and reused multiple times.^[^ ^39^ ^]^ For higher molecular weight and solid HCs, this kind of chemical recycling has already been demonstrated for different standard consumer polymers on an industrial scale.^[^ ^47^ ^]^ As an example for recycling of solid higher molecular weight PEs, PE‐WAX was standardly collected with SPION B and afterwards washed first in 1,2,4‐Trichlorobenzene (TCB) at 120 °C and secondly in n‐hexane. The PE molecules dissolve in TCB while the iron oxide nanoparticles are collected magnetically. This leads to a full recovery of the SPIONs enabling a reusability over at least 3 cycles (s. Figure S5, Supporting Information).

However, most of the recycling processes are time and energy consuming. An alternative sustainable way of handling waste is upcycling. For instance, our collected PE‐SPION‐“waste” can be reused for removing crude oil from a water surface. Crude oil, a mixture of different HCs, has been collected by applying SPIONs only in an efficient and reusable way.^[^ ^39^ ^]^ However, by using the polymer‐SPION aggregates the HCC for oil removal can even be elevated by 54.3% or 63.4%, respectively, in comparison with applying only functionalized SPIONs (s. Figure 3B). The porous structure that consists of hydrophobic PE and SPIONs provides a superior network for viscous crude oil to infiltrate. It also shows that SPION A binds less PE but more crude oil due to the more hydrophobic nature of the uncharged, alkylphosphonic acid. This also leads to a higher overall HCC than SPION B. The collected oil can also be completely washed off by n‐hexane (s. Figure S6, Supporting Information), resulting in PE‐SPION agglomerates, which can again be recycled like described above. This allows a complete sustainability loop consisting of up‐ as well as recycling (s. Figure 3A).

## 3 Conclusion

We have demonstrated that functionalized SPIONs are able to adsorb and to magnetically collect the full range of common HCs from water with only one sorbent platform. The SPION systems perform for liquid n‐alkanes over low molecular PE waxy flakes to solid cross‐linked PE spheres (microplastics). It covers the almost complete bandwidth of molecular weight (C_15_ to C_∞_) but also different morphologies and sizes of HC contaminants from 1.8 nm to 10 µm. The choice of shell molecules enables a tuning of SPION ζ‐potential from negative to positive. The magnetic remediation occurs reliable and the SPIONs can be reused with similar efficiency after recycling. An interesting upcycling method was explored by using the magnetically collected hybrid aggregates of micro‐/nanoplastics and SPIONs as efficient sorbent material to remove mixed liquid HCs (crude oil) from a water surface in a second step. The concept of collecting waste with waste (or short: waste^2^) leads to enhanced collection capabilities of up to 63.4% compared to the use of SPIONs only. In order to further improve the sustainability of the overall process, novel recycling methods need to be developed beyond the existing solvent‐based concepts. However, the combination of broad range activity with simple inexpensive SPION sorbents with the simple magnetic waste separation as well as re‐and upcycling capability makes the concept a unique approach in next‐generation wastewater treatment.

## 4 Experimental Section

### SPION Functionalization

The maghemite nanoparticles (Fe_2_O_3_) were provided by Comar Chemicals (Pty) Ltd and show a diameter of 9.6 nm according to BET measurements assuming spherical‐like shape and an average diameter of 10 nm by HRTEM.^[^ ^40^ ^]^ These nanoparticles (c = 3 mg mL^−1^) as well as the modifying molecules (c = 20 mm), namely n‐Octadecylphosphonic acid (PAC_18_) or (12‐Dodecylphosphonic acid)‐N,N‐dimethyl‐N‐octadecyl ammonium chloride (PAC_12_NC_18_, both bought at SiKÉMIA), were dispersed in methanol. Next, molecule solution and SPION dispersion were mixed in a 3:10 ratio and sonicated for 30 min (Sonocool 255, Bandelin). Afterward, the samples were centrifuged (Multifuge X1R, Heraeus) at 13 000 rpm for 30 min at 5 °C (PAC_12_NC_18_) or 20 min at 20 °C (PAC_18_) and the supernatant was disposed. To remove the unbound phosphonic acids, the functionalized SPIONs were washed two times in methanol and two times in deionized (DI) water. Finally, the residual solvent evaporated overnight at 80 °C.

### SPION and Hydrocarbon Characterization

The functionalization was qualitatively investigated by attenuated total reflectance Fourier transform infrared spectroscopy (ATR‐FTIR, IR Prestige 21, Shimadzu) in a range from 4000–400 cm^−1^ and a resolution of 4 cm^−1^. Thermogravimetric analysis (TG 209 F1 Libra, Netzsch) was utilized to get information about the surface grafting of the functionalized SPIONs. For that, the temperature was gradually increased with 30 K min^−1^ up to an end temperature of 1000 °C with a gas flow consisting of 40 mL min^−1^ nitrogen and 10 mL min^−1^ oxygen. The hydrodynamic diameter and ζ‐potential were determined by dynamic light scattering (Zetasizer Nano ZS, Malvern Panalytical) at specific pH values, which were titrated by hydrochloric acid (HCl) and sodium hydroxide (NaOH).

### Preparation of Hydrocarbon Samples

The n‐alkanes were purchased from Sigma Aldrich. To disperse them in water, the alkane‐water mixture was placed in the oven (80 °C) for one hour to let the alkane melt. Afterward, those solutions were mixed for 7.5 min at 2500 rpm using the SpeedMixer DAC 150 SP (Hausschild SpeedMixer). The PE wax (PE‐WAX) was gratefully provided by DEUREX AG and delivered in a dispersion stabilized by a surfactant. To remove the stabilizer and get the clean PE surface, the wax was washed 5 times in MeOH and another 2 times in DI water. The stabilized PE single crystals (PE‐SC) were synthesized according to literature.^[^ ^48^ ^]^ The stabilized PE‐SC were used as received. The PE micro‐/nanoparticles (PE‐MNP) were purchased from Cospheric LLC as a dry powder. For a 0.1 wt% dispersion, an according amount was wetted with 2‐propanol (Carl Roth), mixed with DI water and sonicated in order to disperse them in water. Finally, the pH of all dispersions was titrated to pH 7 with HCl and NaOH.

The Saharan Blend crude oil was kindly provided by British Petroleum Germany. For sample preparation, 500 µl were pipetted on a water surface.

### Extraction Process and Analysis

For PE and alkanes, 3.5 mg SPIONs were wetted with 50 µl tetrahydrofuran (Carl Roth). Hydrocarbon dispersion of 20 mL was added. While PE‐WAX, PE‐SC and PE‐MNP dispersions were sonicated for 10 min, the alkanes were mixed using a vortex mixer at 2000 rpm to avoid the phase changing influence of temperature. Regarding the oil, 10 mg of SPIONs were placed on the oil‐contaminated water surface and kept moving on a shaking plate (Heidolph Unimax 1010) for 5 min at 200 rpm. Afterward, the SPIONs were magnetically collected for 30 min (alkanes and PE variations) or 10 min (oil), respectively.

The collected oil‐ and alkane‐SPION agglomerates were washed in n‐hexane, the SPIONs were magnetically removed and the hexane solution was qualitatively as well as quantitatively analyzed with gas chromatography‐mass spectrometry (GC‐MS) composed of a gas chromatography GC 2010 Plus using a Supelco Equity‐5 column and a mass spectrometry GC‐MS OP2010 SE. The alkane samples were measured in splitless mode with helium as carrier gas. The injection temperature was 280 °C while the oven temperature was gradually increased from 40 to 300 °C with a rate of 30 °C min^−1^ and a plateau of 2 min at the final temperature. For the oil samples, the oven temperature was increased from 60 to 325 °C with a rate of 80 °C min^−1^ and a plateau of 6 min. Representative spectra are shown in Figure S7 (Supporting Information). For those HCs, the HCC was determined by dividing the mass of collected HCs by the mass of invested iron oxide. In contrast, the PE content of the collected PE‐SPION agglomerates was determined via thermogravimetric analysis using the same conditions as for particle characterization (s. Supporting Information). The HCC for PE‐WAX, PE‐SC and PE‐MNP was calculated according to literature.^[^ ^40^ ^]^ For each hydrocarbon‐SPION combination, at least 6 samples were prepared and analyzed. It must be noted that there was no absolute comparability between the HCC values of n‐alkanes and PE since the quantification method differs.

### Microscopic Analysis

The hydrocarbon‐water dispersions were drop‐cast on a glass substrate for reflected light microscopy (Leica DM2500 M) of C_15_, C_17_ and C_19_. The magnetically collected agglomerates of HC and SPION were transferred from the extraction set up with a spatula to the glass substrate. The scanning electron microscopy (SEM) images (s. Figures 1 and 2) and SEM‐energy dispersive X‐ray spectroscopy (EDXS) maps (s. Figure S3, Supporting Information) of PE‐WAX and its according agglomerates were acquired with a FEI Helios NanoLab 660 Dual Beam SEM‐FIB instrument equipped with an Oxford X‐max 150 mm^2^ EDXS detector. The EDXS maps, which show the net intensities of the K line series of Fe and C, were acquired at an acceleration voltage of 20 kV. The samples were prepared in the same way as described before except that this time a polished aluminum sample holder was used instead of the glass substrate. Additionally, a layer of 2.5 nm platinum‐palladium (80:20) alloy was sputtered (Q150T S, Quorum Technologies) on top of the sample to avoid charge accumulation. The PE‐SC as well as their according aggregate with SPION B, which have been redispersed, were drop‐cast on a silicon nitride window grid and dried to further analyze by transmission electron microscopy (TEM). The high‐angle annular dark‐field scanning TEM (HAADF‐STEM) image (s. Figure 2) and STEM combined with energy dispersive X‐ray spectroscopy (STEM‐EDXS) maps (s. Figure S4, Supporting Information) were acquired with a Spectra 200 transmission electron microscope (Thermo Fisher Scientific) equipped with a Super‐X detector and operated at 200 kV. The PE‐MNP and their SPION B‐aggregates were prepared as the PE‐WAX samples except that here the samples were drop‐cast or transferred on a silicon wafer, which was attached to the sample holder. The images were acquired with a Zeiss AURIGA featuring a GEMINI column.

### Recycling

The PE‐WAX‐SPION agglomerates were washed in 1,2,4‐trichlorobenzene (TCB) at 120 °C. Afterward, the particles were magnetically collected and rinsed with hexane to remove coarse residues of TCB. Next, the particles were washed in hexane using an ultrasonic bath (Sonocool 255, Bandelin) for 10 min. Finally, the particles were magnetically collected and dried before reuse.

## Conflict of Interest

The authors declare no conflict of interest.

## Supporting information



Supporting Information

## Data Availability

The data that support the findings of this study are available from the corresponding author upon reasonable request.
