# Supplementary material for: A Sustainable Method for Removal of the Full Range of Liquid and Solid Hydrocarbons from Water Including Up‐ and Recycling
Source: Adv Sci (Weinh). 2023 Oct 9;10(32):2302495. doi: 10.1002/advs.202302495 (PMC10646276; doi:10.1002/advs.202302495)
Supplement: Supplementary file 1 — Supporting Information [file ADVS-10-2302495-s001.pdf]

ADVANCED SCIENCE
Open Access

Supporting Information

for *Adv. Sci.*, DOI 10.1002/advs.202302495

A Sustainable Method for Removal of the Full Range of Liquid and Solid Hydrocarbons from Water Including Up- and Recycling

*Henrik Gaß, Marco Sarcletti, Lukas Müller, Sabine Hübner, Tadahiro Yokosawa, Hyoungwon Park, Thomas Przybilla, Erdmann Spiecker and Marcus Halik**

Supporting Information

A Sustainable Method for Removal of the Full Range of Liquid and Solid Hydrocarbons from Water with Up- and Recycling Options

*Henrik Gaß, Marco Sarcletti, Lukas Müller, Sabine Hübner, Tadahiro Yokosawa, Hyoungwon Park, Thomas Przybilla, Erdmann Spiecker, Marcus Halik**

1. SPION and hydrocarbon characterization

The functionalized iron oxide nanoparticles were analyzed by attenuated total reflectance Fourier transform infrared spectroscopy (ATR-FTIR). Both the alkyl peaks (2920 cm^{-1} and 2850 cm^{-1}) and the phosphonic acid peaks (1150 and 850 cm^{-1}) from the molecule were transferred to the particle (s. **Figure S1**A). ATR-FTIR spectra of the hydrocarbon variants show common traits: peaks at 2920 cm^{-1}, 2850 cm^{-1}, 1470 cm^{-1} and 720 cm^{-1} are typical for CH_2-CH_2 and CH_3 bindings (s. **Figure S2**A).

Thermogravimetric analysis (TGA) revealed an increased mass loss (10.6 % for SPION A and 6.6 % for SPION B) compared to the pristine ones (2.8 %) which indicates a gain in organic compounds on the surface during functionalization (s. Figure S1B). The fact that SPION B shows less mass loss despite of $PAC_{12}NC_{18}$ having a higher molar mass (582.33 g mol^{-1}) than PAC_{18} (334.47 g mol^{-1}) can be explained by electrostatic repulsion between $PAC_{12}NC_{18.}$ This hinders the molecules to densely pack on the surface leading to less organic compounds on the SPIONs and therefore, less mass loss during TGA.

The hydrodynamic diameter of the hydrocarbon variants determined by dynamic light scattering (DLS) differs significantly while the ζ-potential is in a similar negative range for all of them (s. Figure S2B and S2C). The ζ-potential of the functionalized SPIONs revealed the main difference between both systems. SPION A covered with the uncharged molecule has a negative ζ-potential at pH 7 (ζ = - 13.7 ± 6.9 mV) while SPION B shows a positive ζ-potential of +23.0 ± 8.5 mV (s. Figure S1C).

The crystalline structure is not altered by the functionalization process as confirmed by high resolution transmission electron microscopy.[1]

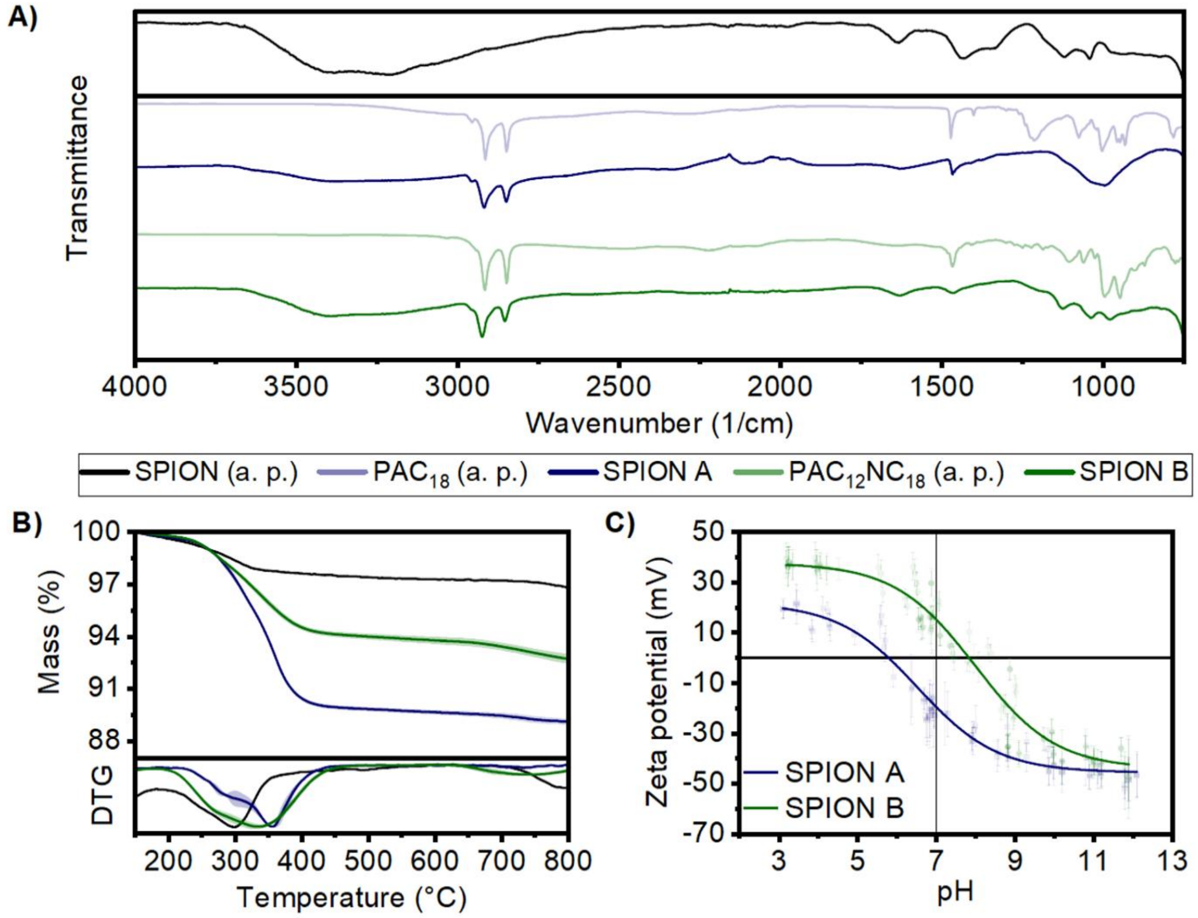

Figure S1: Characterization of functionalized SPIONs: A) ATR-FTIR spectra show bound phosphonic acids on the particles' surface. B) TGA shows a higher mass loss for functionalized SPIONs indicating a successful functionalization. C) The transparent dots are the measured values, the line shows an according Boltzmann fit (R^2 = 0.97 for SPION A, R^2 = 0.94 for SPION B). This measurement revealed that SPION A has a negative and SPION B a positive ζ-potential a pH 7.

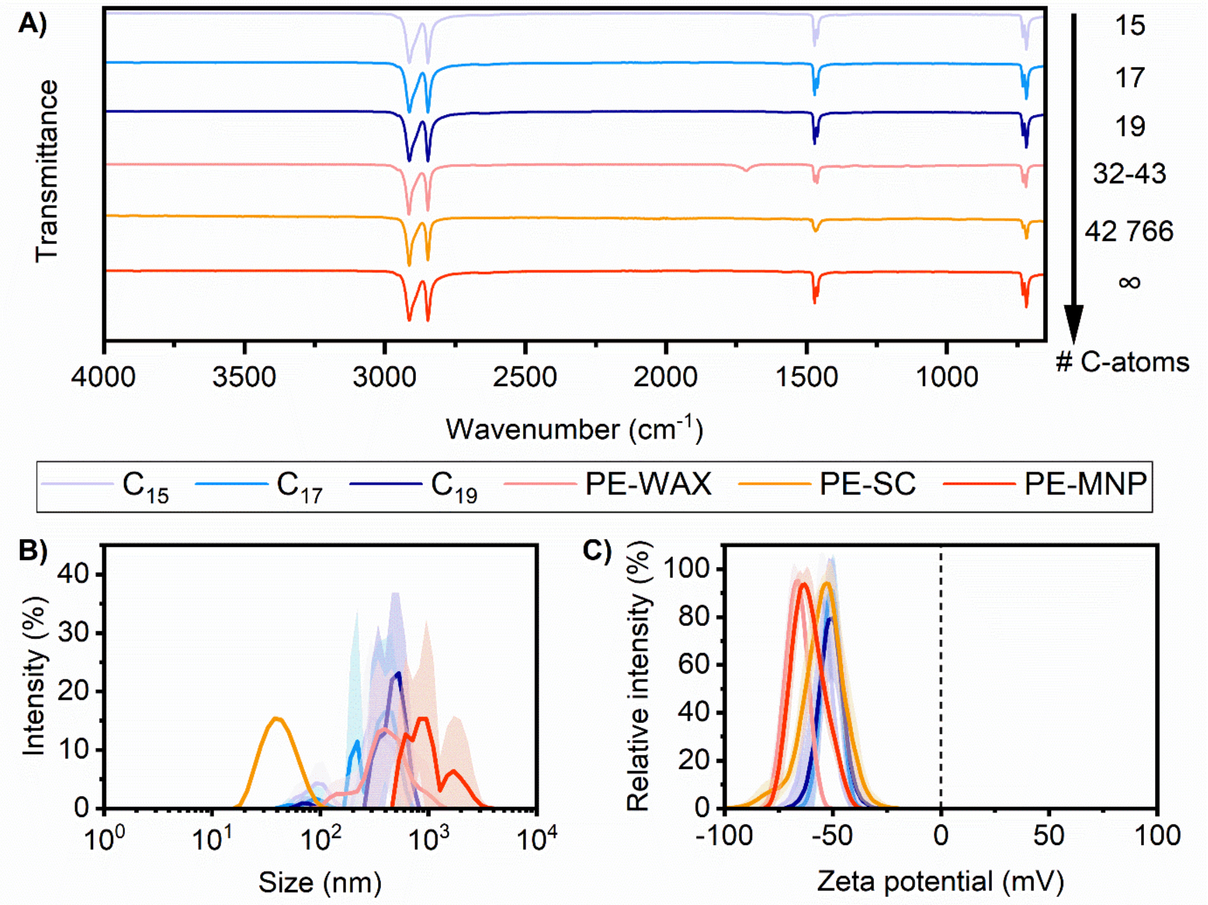

Figure S2: Characterization of hydrocarbons: all show the same fundamental structure (A) and similar ζ-potential (C) but differ in size (B) and morphology.

2. SEM and TEM analysis

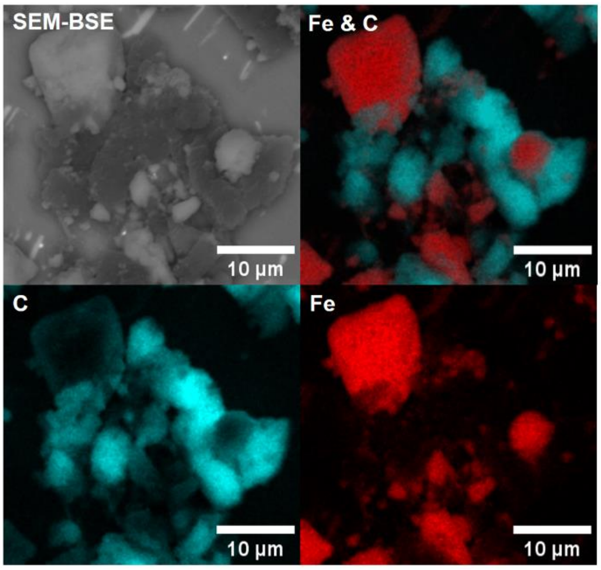

Figure S3: A scanning electron microscopy (SEM) image (backscattered electrons (BSE), top left) and the according elemental mapping by energy dispersive X-ray spectroscopy (EDXS) show the success of extraction. It reveals the distribution of the iron (red signal) of the SPIONs which agglomerates with the PE-WAX (cyan signal)

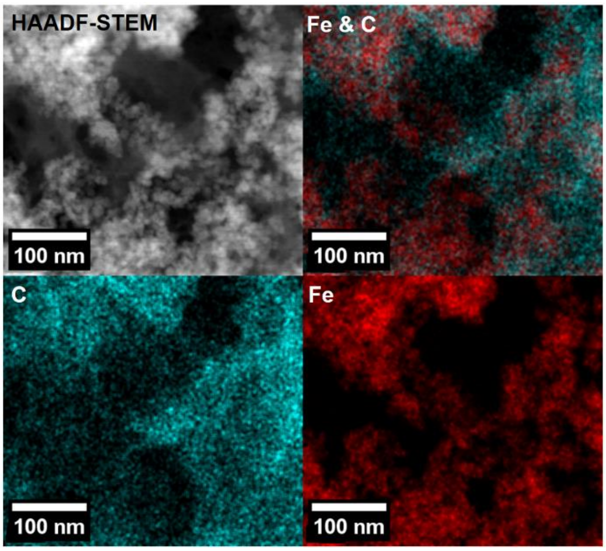

Figure S4: A high-angle annular dark field image (HAADF, top left) and an elemental mapping by EDXS imaged in a scanning transmission electron microscope (STEM) show the morphology and composition of extracted aggregates. Areas with a high carbon signal (net intensities) (cyan) but low iron signal (red) correspond to the flat polyethylene single crystals proving the extraction success.

3. Recycling

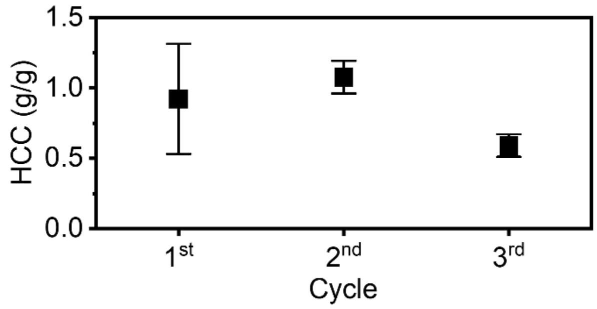

Figure S5: Even after a chemical treatment to remove the extracted PE-WAX, SPION B is still able to collect PE-WAX without significant losses as the HCC after the 3rd cycle is still within the error bars of the 1st cycle.

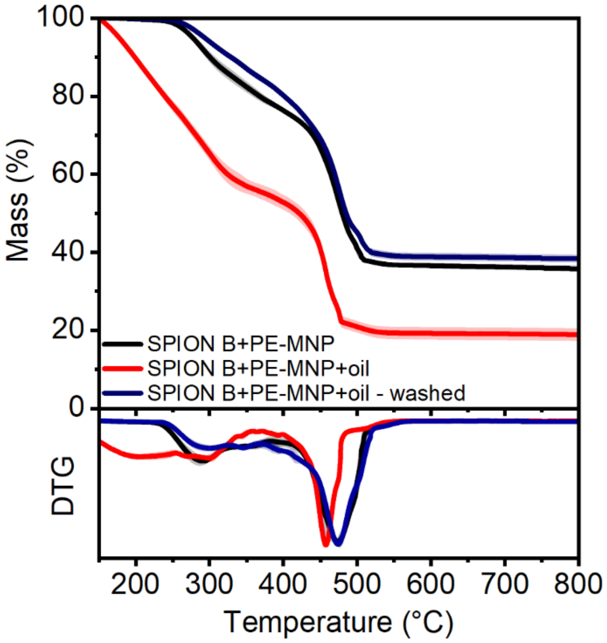

Figure S6: Thermogravimetric analysis data is shown for PE-MNP collected by SPION B (black). These agglomerates were used as a sorbent for crude oil (red) which was completely washed off by n-hexane (blue) as the mass loss comply with that of unused PE-MNP-SPION agglomerates (black).

4. Gas chromatography spectra

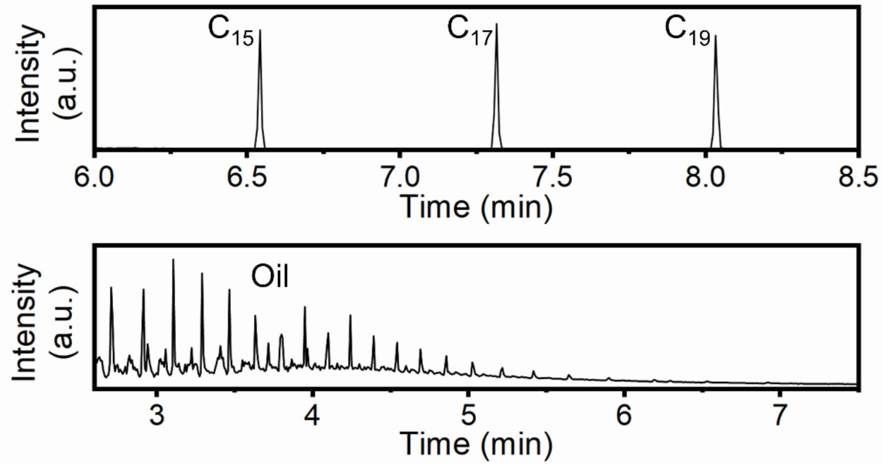

Figure S7: Representative gas chromatography spectra of n-alkanes (top) and crude oil (bottom).

References

[1] M. Sarcletti, H. Park, J. Wirth, S. Englisch, A. Eigen, D. Drobek, D. Vivod, B. Friedrich, R. Tietze, C. Alexiou, D. Zahn, B. Apeleo Zubiri, E. Spiecker, M. Halik, *Mater. Today* **2021**, *48*, 38.
